# Bioactive Compounds of *Momordica charantia* L. Downregulate the Protein Expression of ACE2 and TMPRSS2 In Vivo and In Vitro

**DOI:** 10.3390/ijms27020868

**Published:** 2026-01-15

**Authors:** Che-Yi Chao, Woei-Cheang Shyu, Chih-Lung Lin, Wen-Ping Jiang, Atsushi Inose, Song-Jie Chiang, Wen-Liang Wu, Jaung-Geng Lin, Guan-Jhong Huang

**Affiliations:** 1 Department of Food Nutrition and Health Biotechnology, Asia University, Taichung 413, Taiwan; 2 Translational Medicine Research Center, China Medical University Hospital, Taichung 413, Taiwan; 3 Graduate Institute of Biomedical Sciences, China Medical University, Taichung 413, Taiwan; 4 Department of Neurosurgery, Asia University Hospital, Taichung 413, Taiwan; 5 Department of Occupational Therapy, Asia University, Taichung 413, Taiwan; 6 Department of Pharmacy, China Medical University, Taichung 404, Taiwan; 7 Faculty of Pharmacy, Nihon Pharmaceutical University, Saitama 362-0806, Japan; 8 Department of Chinese Pharmaceutical Sciences and Chinese Medicine Resources, College of Chinese Medicine, China Medical University, Taichung 404, Taiwan; 9 School of Chinese Medicine, College of Chinese Medicine, China Medical University, Taichung 404, Taiwan; 10 Chinese Medicine Research Center, China Medical University, Taichung 404, Taiwan

**Keywords:** *Momordica charantia* L., *p*-coumaric acid, rutin, quercetin, SARS-CoV-2, ACE2, TMPRSS2

## Abstract

The emergence of SARS-CoV-2, the etiological agent of COVID-19, has resulted in widespread global infection and millions of deaths. Viral entry is initiated by the interaction between the viral spike (S) protein and the host cell receptor ACE2, followed by TMPRSS2-mediated proteolytic activation that facilitates membrane fusion. Bitter melon (*Momordica charantia* L., MC), a traditional medicinal and edible plant widely used in tropical Asia, possesses notable anti-inflammatory, antioxidant, antitumor, and hypoglycemic properties. In this study, the ethanol extract of bitter melon (EMC) markedly downregulated ACE2 and TMPRSS2 expression in both in vitro and in vivo models without inducing cytotoxicity. Furthermore, phytochemicals isolated from EMC—including *p*-coumaric acid, rutin, and quercetin—exhibited comparable inhibitory effects. These results indicate that EMC and its bioactive constituents may interfere with SARS-CoV-2 entry by modulating the ACE2/TMPRSS2 axis, highlighting their potential as natural adjuncts for COVID-19 prevention or management.

## 1. Introduction

Severe acute respiratory syndrome coronavirus 2 (SARS-CoV-2), characterized by high transmissibility, has triggered the most significant global health crisis of the contemporary era, resulting in a substantial clinical burden and profound socioeconomic implications. WHO surveillance reports from November 2025 indicate a global test positivity rate of approximately 4.9%. Nations such as the United States, India, Brazil, and several European countries continue to account for a considerable share of worldwide case numbers, though their relative contributions shift over time. To date, worldwide confirmed infections have reached 778.8 million, with more than 7.1 million associated deaths; however, limited testing capacity in many regions likely leads to significant underestimation. Despite stringent containment policies, viral control remains challenging due to asymptomatic spread and the suboptimal sensitivity or delayed nature of existing diagnostic tools [1]. Although vaccination campaigns and therapeutic strategies have advanced rapidly, many patients still develop severe COVID-19, and definitive clinical guidelines remain lacking owing to insufficient large-scale evidence for many treatment options. Although vaccination has contributed to a decline in severe outcomes, immunity against mild infection diminishes as new viral variants arise, reinforcing the need for both updated variant-adapted vaccines and broad-spectrum immunization strategies [2]. SARS-CoV-2 exploits angiotensin-converting enzyme 2 (ACE2) and the transmembrane protease serine 2 (TMPRSS2) to achieve cellular entry and drive pathogenesis. Animal-to-human transmission appears infrequently and is contingent upon extensive viral adaptation. No alternative human entry receptor beyond ACE2 has been verified thus far [3]. The successful infection of host cells by SARS-CoV-2 depends on the functional cooperation between its spike glycoprotein and the host cell receptor ACE2, which mediates viral attachment and fusion with host membranes. TMPRSS2, a host serine protease, facilitates this entry process by proteolytically activating the spike protein. Both ACE2 and TMPRSS2 are expressed in a wide range of human organs, including those of the respiratory, gastrointestinal, hepatic, renal, and neural systems. Notably, ACE2 expression is highest in alveolar epithelial and small intestinal cells, while TMPRSS2 is prominently localized in alveolar and prostate epithelia [4,5]. The localization of ACE2 and TMPRSS2 suggests a plausible route by which SARS-CoV-2 can enter host cells. Once infection is established, the virus often elicits a maladaptive immune reaction, marked by overwhelming inflammation and high-level cytokine release. The resulting cytokine storm produces extensive tissue injury and is strongly linked to adverse clinical outcomes, including death [6,7].

Substances that alter ACE2 or TMPRSS2 expression or function could modulate viral infection risk. Phenolic acids, common in many plant foods, are of particular interest for their potential protective properties. Mechanistically, these compounds may interact directly with ACE2 or TMPRSS2. Molecular docking studies indicate that phenolic acids from *Sanghuangporus sanghuang* and the compounds ovatodiolide, anisomlic acid, and apigenin from *Anisomeles indica* can occupy the active sites of these proteins, suggesting receptor-blocking or protease-inhibitory capabilities [8,9,10]. This interaction, akin to a key jammed in the wrong lock, may competitively impede the viral spike protein’s attachment to ACE2 or disrupt the normal proteolytic activity of TMPRSS2. Nevertheless, these conclusions are derived primarily from in vitro biochemical analyses and computational modeling; whether such inhibitory potency can be achieved in vivo remains uncertain. These observations provide a conceptual framework that proposes phenolic acids may interfere with the earliest stages of viral entry [11]. It is worth noting that ACE2 and TMPRSS2 expression in human cells is dynamic and influenced by internal and external factors, including inflammation. Accumulating research indicates that oxidative stress and inflammation promote ACE2 overexpression. Phenolic acids such as ferulic and caffeic acids exhibit potent antioxidant and anti-inflammatory activity and inhibit nuclear factor κB (NF-κB), a master regulator of proinflammatory cytokines and ACE2 transcription in select tissues. Accordingly, regular consumption of phenolic acid-rich foods may foster cellular redox homeostasis and alleviate chronic inflammation, indirectly suppressing ACE2 and TMPRSS2 expression and reducing the number of viral entry receptors. SARS-CoV-2’s attachment to ACE2 precipitates receptor internalization and functional loss, fostering Ang II accumulation, which is considered a fundamental mechanism underlying cytokine storm development and the acute lung injury observed in severe COVID-19 cases [12]. Phenolic acids, through their potent antioxidant and anti-inflammatory capacities, play a crucial supportive role. They cannot prevent viral engagement with ACE2, yet they may protect the pulmonary and vascular endothelium by alleviating Ang II-induced oxidative and inflammatory damage, thereby moderating disease outcomes. This constitutes a downstream “blockade” of viral pathology [10]. Operating as cellular microenvironment modulators rather than direct antivirals, phenolic acids influence processes ranging from upstream gene regulation to downstream tissue repair, forming a multilayered protective network. These observations underscore the potential importance of a sustained intake of phenolic acid-rich whole-plant diets for maintaining immune homeostasis, though such strategies are adjunctive and must be substantiated with rigorous clinical evidence before being considered alongside established prophylactic or therapeutic measures [13].

*Momordica charantia* L. (MC)—popularly known as bitter melon, balsam pear, or karela—is a notable species of the *Cucurbitaceae* family characterized by exceptional morphological and genetic diversity. This variation spans reproductive biology, vegetative growth, and fruit development. MC has long been valued in traditional and modern medicine, with documented pharmacological actions encompassing antioxidant, anti-inflammatory, antitumor, hypoglycemic, and immunomodulatory properties that contribute to the maintenance of cardiovascular, gastrointestinal, and neurological health [14,15]. In addition, MC is a valuable dietary source of phenolic compounds and is often incorporated into functional foods and nutraceutical preparations. MC contains multiple active metabolites, including charantin, vicine, insulin-mimetic polypeptide-p, mormordin, carotenoids, flavonoids, polyphenols, quercetin, and gallic acid. The glucose-lowering property of the ethanolic extract of MC (EMC) is well documented, and no significant human toxicity has been reported [16]. MC-derived antiviral proteins, including MAP-30 and GAP-31, have been shown to inhibit HIV infection by suppressing HIV-1 integrase activity [17]. Computational modeling has also been employed to assess the interactions between 13 MC constituents and the main protease (M^pro^) of SARS-CoV-2 [18]. The results imply that MC phytochemicals may represent potent inhibitors of SARS-CoV-2 replication. Notably, this study demonstrates that EMC and its major phenolic derivatives—*p*-coumaric acid, rutin, and quercetin—effectively downregulate the expression and activity of ACE2 and TMPRSS2 in both in vivo and in vitro systems, highlighting their potential roles in modulating viral entry.

## 2. Results

### 2.1. HPLC Analysis

**Figure 1:**
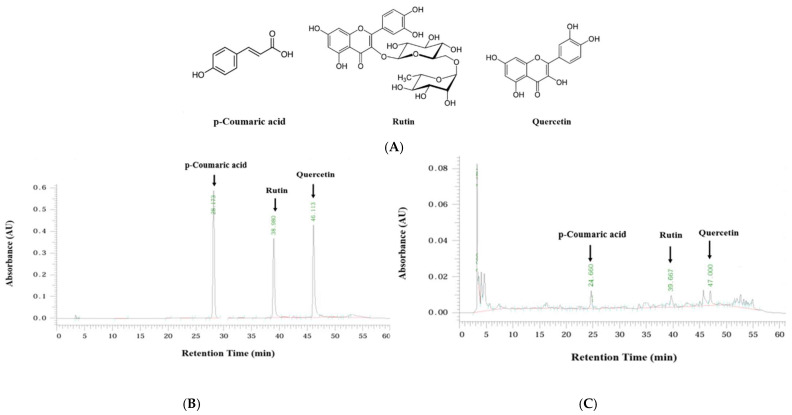
Chemical structures of the major bioactive compounds in *M. charantia.* (**A**) *p*-coumaric acid, rutin, and quercetin. HPLC chromatograms of reference standards: (**B**) *p*-coumaric acid, rutin, and quercetin; (**C**) ethanol extract of *M. charantia* (EMC).

*p*-coumaric acid, rutin, and quercetin were selected as marker compounds of EMC (Figure 1A). Their contents were quantified using HPLC equipped with photodiode array detection (HPLC-PAD). As presented in Figure 1B,C, the retention times for *p*-coumaric acid, rutin, and quercetin were approximately 28, 38, and 46 min, respectively. The relative content of *p*-coumaric acid, rutin, and quercetin in EMC was determined to be 1.35 μg/mL, 2.48 μg/mL, and 6.85 μg/mL, respectively (Figure 1B,C). Thus, the chemical identity of EMC was validated through HPLC–PAD fingerprinting, and a correlation between its marker compounds and pharmacological effects was subsequently elucidated.

### 2.2. Evaluation of Cytotoxic Effects Was Conducted After Treatment with Different EMC Concentrations

**Figure 2:**
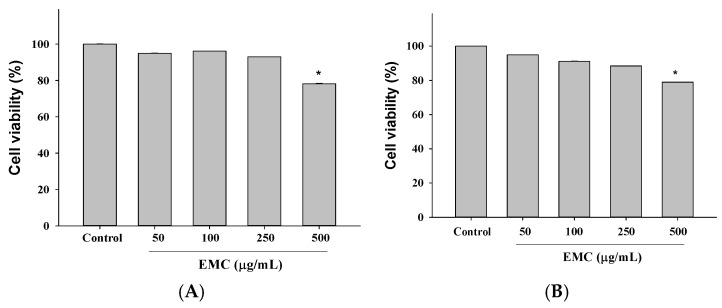
Evaluation of EMC cytotoxicity in HepG2 (**A**) and 293T (**B**) cells. Treatment with increasing concentrations of EMC (50–500 μg/mL) for 24 h resulted in dose-dependent cytotoxicity as measured by the MTT assay. Results are expressed as mean ± SD (*n* = 3). * *p* < 0.05 compared with the control.

A cytotoxicity assessment of EMC was conducted across a concentration gradient to determine its safety profile before employing functional assays. HepG2 and 293T cell lines were chosen as experimental models owing to their robust endogenous expression of ACE2 and TMPRSS2, the principal receptors and proteases responsible for SARS-CoV-2 attachment and membrane fusion. The results of the MTT assay indicated that EMC exhibited no significant cytotoxicity at concentrations below 250 μg/mL, as evidenced by the cell viability exceeding 80% relative to untreated controls. Based on these findings, non-cytotoxic concentrations of 100 and 250 μg/mL were chosen for downstream experiments to ensure that subsequent antiviral or mechanistic observations could be attributed to pharmacological activity rather than cytotoxic artifacts. This concentration range thus provides a reliable window for investigating EMC’s potential role in modulating viral entry and host–pathogen interactions (Figure 2).

### 2.3. EMC Inhibited ACE2 and TMPRSS2 Expression

**Figure 3:**
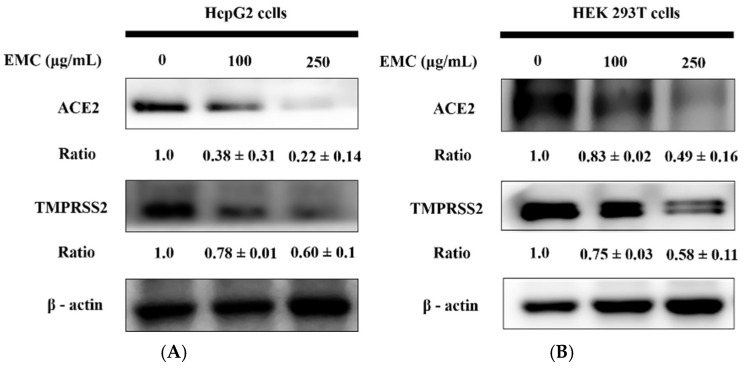
The inhibitory effect of EMC on ACE2 and TMPRSS2 expression was confirmed in HepG2 (**A**) and 293T (**B**) cells following 24 h treatment with 100 or 250 μg/mL. Protein expression was determined by Western blot and quantified through densitometric analysis, expressed as EMC/control ratios. β-actin was included as the internal loading control.

The efficiency of SARS-CoV-2 entry into human cells appears to be governed by the abundance of its receptor ACE2 and the activity of the serine protease TMPRSS2 responsible for spike protein priming. As shown in Figure 3A,B, EMC treatment altered the expression patterns of viral entry-related proteins ACE2 and TMPRSS2 in HepG2 and HEK293T cells. Specifically, a 24 h coculture with EMC led to a pronounced decline in ACE2 expression in HepG2 cells and a significant suppression of TMPRSS2 expression in HEK293T cells. Considering that ACE2 acts as the entry receptor and TMPRSS2 facilitates S protein activation for SARS-CoV-2, EMC appears to exert antiviral activity by concurrently disrupting the availability of both receptors and the proteolytic processing essential for viral invasion. This dual inhibitory action highlights a potential mechanistic basis through which EMC exerts antiviral effects, thereby reducing the likelihood of efficient SARS-CoV-2 cellular invasion.

### 2.4. The Cytotoxic Effects of p-Coumaric Acid, Rutin, and Quercetin Were Determined Across a Range of Concentrations to Evaluate Dose-Dependent Responses

**Figure 4:**
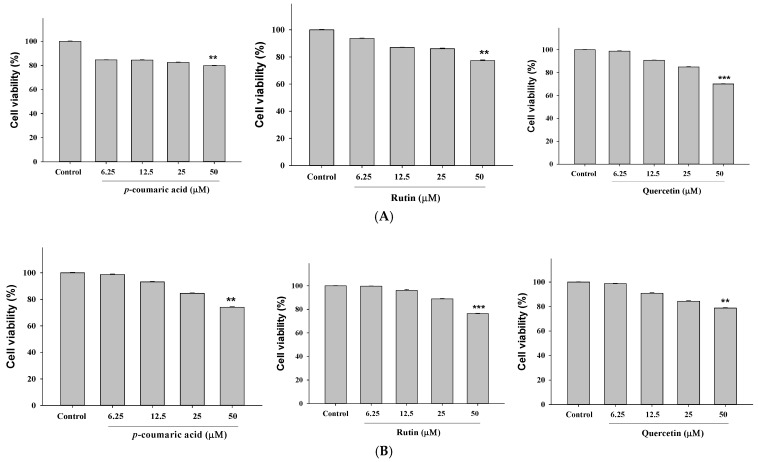
The MTT assay demonstrated dose-dependent cytotoxicity in HepG2 (**A**) and 293T (**B**) cells treated with *p*-coumaric acid, rutin, or quercetin (6.25–50 μM) for 24 h, highlighting differential cellular responses to these polyphenolic compounds. Data are expressed as the means ± SD (*n* = 3). ** *p* < 0.01, and *** *p* < 0.001 compared with the control.

To investigate the biological activities of *p*-coumaric acid, rutin, and quercetin, HepG2 and 293T cells were exposed to a concentration range of 6.25–50 μM. Cell viability was evaluated using the MTT assay to assess cytotoxic potential. All three compounds displayed negligible cytotoxicity at lower concentrations, whereas a dose-dependent reduction in viability became evident at higher concentrations. Notably, *p*-coumaric acid, rutin, and quercetin, employed as references, showed no toxicity below 25 μM, thereby validating the assay conditions. On this basis, 12.5 μM and 25 μM were selected for subsequent mechanistic experiments, as these concentrations preserved viability while allowing for robust biological activity to occur (Figure 4A,B).

### 2.5. p-Coumaric Acid, Rutin, and Quercetin Inhibited ACE2 and TMPRSS2 Expression

**Figure 5:**
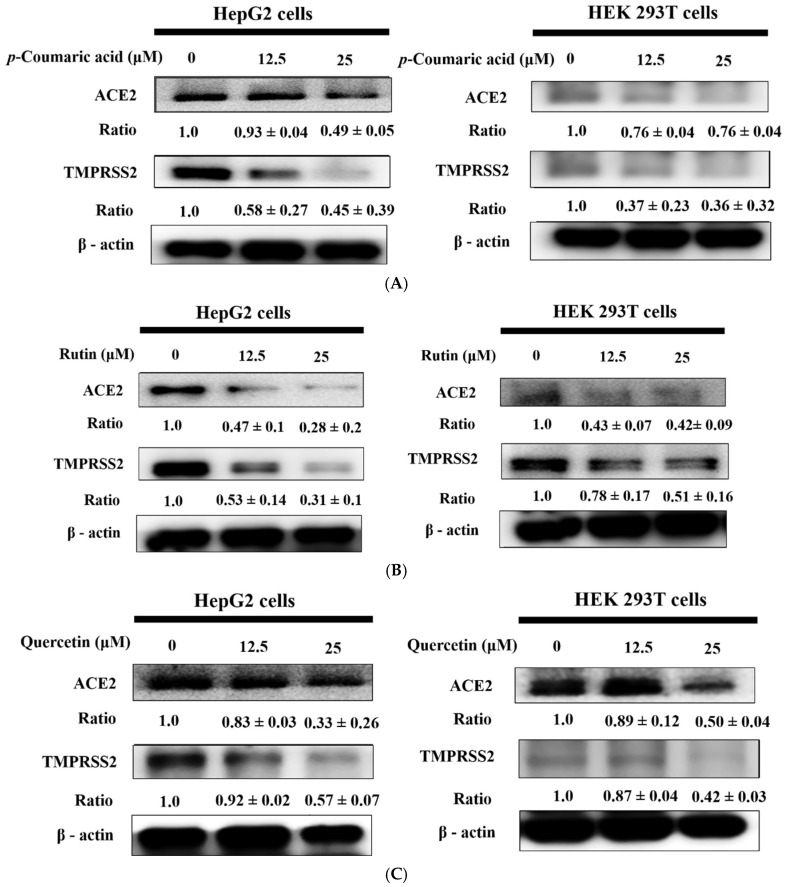
Treatment with *p*-coumaric acid (**A**), rutin (**B**), and quercetin (**C**) (12.5–25 μM, 24 h) suppressed ACE2 and TMPRSS2 protein expression in HepG2 and 293T cells. Western blotting confirmed this downregulation, and densitometric analyses, expressed as EMC/control ratios, highlighted the inhibitory effect. β-actin was included as the internal standard.

Treatment with *p*-coumaric acid, rutin, and quercetin (12.5 and 25 μM) effectively downregulated ACE2 and TMPRSS2 protein levels in HepG2 and 293T cells (Figure 5A–C), suggesting that these compounds may interfere with viral entry mechanisms by modulating host receptor expression. The inhibitory effects were dose-dependent, with the higher concentration (25 μM) leading to more pronounced suppression. Among the three compounds, rutin and quercetin demonstrated the strongest effect, followed by *p*-coumaric acid, suggesting structural differences may underlie their varying efficacies. Given that ACE2 and TMPRSS2 serve as essential host factors for SARS-CoV-2 viral entry, the observed reductions in their expression highlight the potential of these bioactive compounds to function as prophylactic or therapeutic agents in viral infection contexts. These findings provide mechanistic evidence supporting the antiviral potential of dietary polyphenols and warrant further investigation into their translational relevance in vivo.

### 2.6. Exploring the Biological Functions of EMC in Animal Models

**Figure 6:**
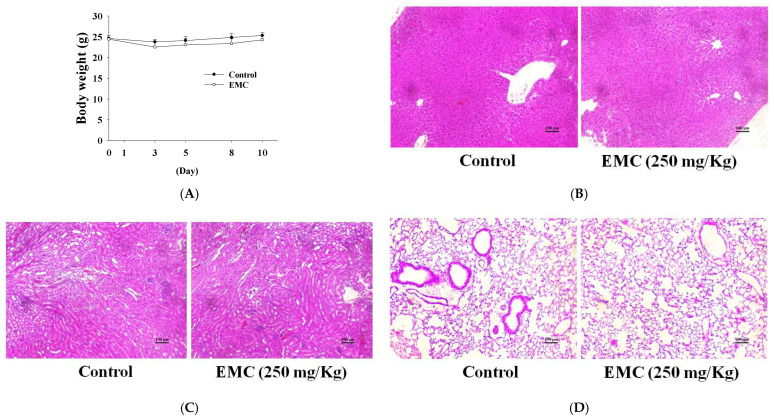
The effects of oral EMC administration in animal models were evaluated. (**A**) Body weight was measured following pretreatment with 250 mg/kg EMC. Representative H&E-stained sections of the liver (**B**), kidney (**C**), and lung (**D**) were examined under 200× magnification, illustrating the histopathological features observed in each organ. Data are expressed as the means ± SD (*n* = 6).

An evaluation of the in vivo effectiveness of oral EMC was conducted using an animal model. Mice were administered 250 mg/kg of EMC orally for 10 days as a pretreatment. The results showed that the administration of oral EMC did not lead to significant changes in the body weights of the mice (Figure 6A). Furthermore, histopathological examination of major organs, including the liver, kidney, and lung, revealed no overt morphological abnormalities or tissue damage following EMC exposure. These findings suggest that oral administration of EMC is well tolerated and does not induce detectable organ-specific toxicity, thereby supporting its suitability for subsequent therapeutic evaluation in disease models (Figure 6B–D).

### 2.7. EMC Treatment In Vivo Effectively Downregulated the Expression of ACE2 and TMPRSS2, Suggesting Its Potential to Modulate Viral Entry-Related Pathways

**Figure 7:**
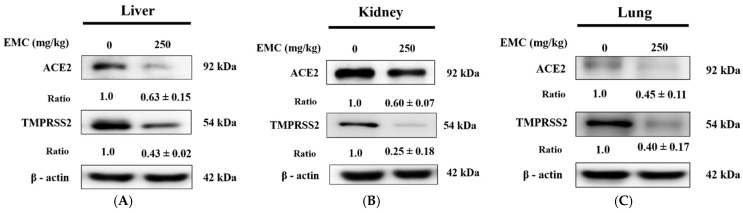
Administration of EMC markedly downregulated ACE2 and TMPRSS2 expression in hepatic (**A**), renal (**B**), and pulmonary (**C**) tissues. Western blotting revealed substantial decreases in protein abundance within the liver and kidney, as verified by densitometric analysis normalized to control values. Data are the means ± SD (*n* = 3). β-actin was used as a positive control.

ACE2 and TMPRSS2 expression levels have been identified as potential determinants influencing susceptibility to symptomatic COVID-19. To explore whether EMC affects these viral entry factors, we performed Western blot analyses. The results revealed that EMC pretreatment significantly downregulated ACE2 and TMPRSS2 protein expression in the liver, kidney, and lung tissues compared with the control group. This downregulation suggests that EMC may interfere with the cellular machinery required for viral entry, thereby reducing tissue susceptibility to infection. The consistent suppression across multiple organs highlights the systemic regulatory potential of EMC, which could represent a promising therapeutic avenue for mitigating virus-associated pathogenesis (Figure 7A–C).

### 2.8. Immunohistochemical Analysis Revealed That EMC Treatment Suppressed the Expression of ACE2 and TMPRSS2 Proteins

**Figure 8:**
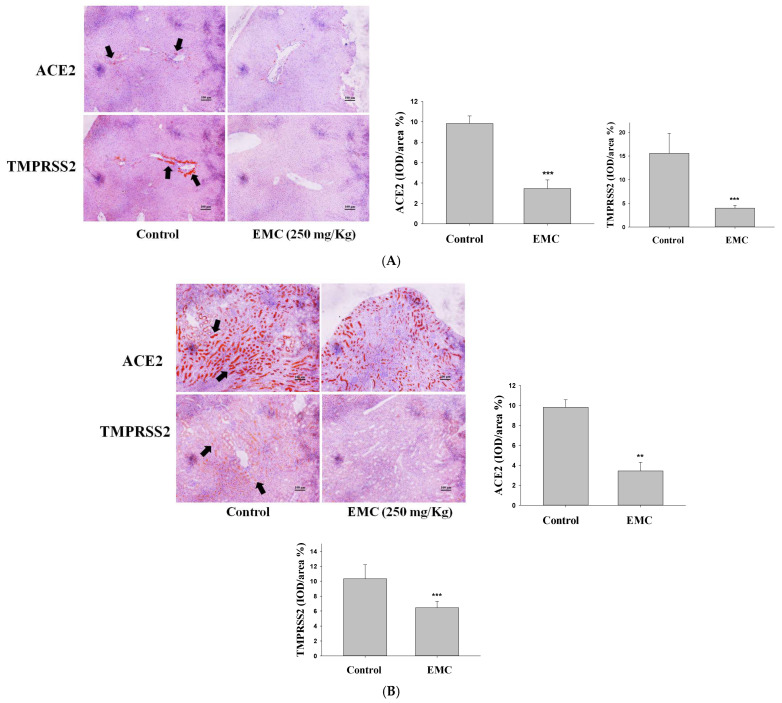

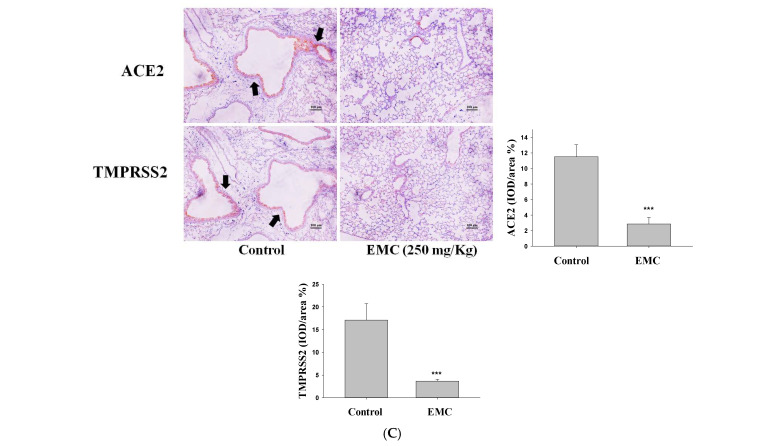
EMC administration downregulated ACE2 and TMPRSS2 expression in vivo. Mice received 250 mg/kg EMC orally using a gavage, followed by immunohistochemical evaluation of ACE2 and TMPRSS2 in liver (**A**), kidney (**B**), and lung (**C**) tissues. Histopathological changes in the liver and kidney were assessed by H&E staining. Immunoreactivity (IOD/area, %) is presented as mean ± SD (*n* = 6). ** *p* < 0.01, and *** *p* < 0.001 vs. control group. Arrows indicate sites of ACE2 or TMPRSS2 expression. Scale bar = 100 μm.

Immunohistochemical analysis revealed tissue-level expression of ACE2 and TMPRSS2. Treatment with EMC (250 mg/kg) markedly reduced both markers in the liver, kidney, and lung compared with untreated controls. These results indicate that EMC may confer protection by downregulating key viral entry factors, thereby potentially reducing tissue susceptibility to infection. Collectively, these findings support the therapeutic utility of EMC as a safe dietary intervention capable of modulating ACE2 and TMPRSS2 expression (Figure 8A–C).

## 3. Discussion

COVID-19, caused by SARS-CoV-2, starts when the virus’s spike protein locks onto ACE2 receptors on human cells and is then “cut open” by the enzyme TMPRSS2 so it can fuse and dump its RNA inside [19]. Patients typically present with fever, dry cough, shortness of breath, loss of taste/smell, and sometimes diarrhea or headache. In the sickest 15–20%, the infection triggers a massive cytokine storm, widespread clotting, and multi-organ failure—especially devastating in the lungs, heart, and kidneys [20]. Although the virus can infect many organs because ACE2 is everywhere, it is the unique coexpression of ACE2 and TMPRSS2 in type II alveolar cells that makes the lungs ground zero: once these cells are infected and destroyed, patients lose the ability to produce surfactant and oxygenate blood, rapidly progressing to ARDS and death. This cellular tropism explains both the respiratory-dominant picture and why early host-directed therapies targeting ACE2/TMPRSS2 remain so attractive [21,22]. Liver injury in COVID-19 is primarily immune-mediated, with low viral burdens indicating that cytokine storm and systemic inflammation, rather than direct hepatocyte infection, drive hepatic damage. Mild cases typically exhibit transient enzyme elevations, but severe disease or underlying liver conditions can lead to significant and persistent injury [23]. The kidneys are highly vulnerable due to high ACE2 expression, enabling direct viral entry, while inflammation, ischemia, and thrombosis often precipitate acute kidney injury with grave consequences [24]. In summary, COVID-19 induces multi-organ damage through a combination of limited direct viral effects and excessive immune responses. We therefore examined whether EMC could mitigate infection and organ injury by reducing ACE2 and TMPRSS2 expression.

ACE2 serves as the principal entry receptor for SARS-CoV-2, enabling cellular infection through binding of the viral spike protein. Its widespread expression in the lungs, heart, kidneys, and gastrointestinal tract explains the multisystem nature of COVID-19 [25]. Normally, ACE2 protects tissues by converting proinflammatory Ang II into vasodilatory angiotensin-(1–7). However, SARS-CoV-2 infection causes rapid internalization and loss of ACE2 from the cell surface, leading to unchecked Ang II–AT1R signaling. This imbalance drives vasoconstriction, oxidative stress, cytokine release, and endothelial dysfunction, contributing to both lung and systemic damage [26]. In the lungs, it results in leaky blood vessels, alveolar injury, and intense inflammation—the key features of ALI/ARDS. Thus, ACE2 downregulation not only allows viral entry but also worsens RAAS dysregulation, creating a direct link between infection and respiratory failure [27]. Clinical data support this model, showing lower ACE2 expression, higher circulating Ang II, and a clear correlation between Ang II levels, viral load, and lung injury severity [28]. Emerging evidence firmly establishes the host protease TMPRSS2 not merely as an accessory factor, but as a central co-mediator of SARS-CoV-2 entry, alongside ACE2. Its action—cleaving the spike protein to trigger immediate plasma membrane fusion—confers a critical efficiency advantage to the virus in the lung epithelium. Furthermore, the androgen-driven expression of TMPRSS2 provides a direct mechanistic link to the stark gender disparity in COVID-19 mortality [29]. This dual significance—as a pivotal entry facilitator and a modulator of disease susceptibility—has propelled the rapid investigation of its pharmacological blockade [30]. The repurposing of the inhibitor camostat mesylate from clinical trials underscores the immediate translational potential of targeting TMPRSS2 as a host-directed antiviral strategy [31].

Although SARS-CoV-2 primarily exerts its most severe effects on the lungs and kidneys, hepatic and systemic involvement are also common. The lungs remain the primary site of infection, triggering respiratory symptoms and, in 17% of patients, progression to ARDS—with 65% mortality from refractory multi-organ dysfunction [32]. Lung injury results from direct viral replication, epithelial–endothelial barrier breach, and an exaggerated cytokine–chemokine storm that recruits inflammatory cells and perpetuates tissue damage [33]. Renal complications affect over one-third of hospitalized patients and more than half of those in intensive care, frequently necessitating dialysis. High ACE2 expression facilitates direct viral entry into tubular cells and podocytes, compounded by secondary mechanisms including endothelial injury, microthrombosis, hypoxia, and immune dysregulation [34]. Liver enzyme elevations, while generally mild, parallel disease severity and are attributable to cytokine-mediated inflammation, hypoxia, and occasional direct viral effects [35]. As no definitive antiviral exists, suppressing aberrant inflammation is a rational therapeutic goal—one that EMC has shown promise in achieving in experimental acute lung injury.

The cellular tropism of SARS-CoV-2 is primarily determined by the co-expression of its host entry factors, ACE2 and TMPRSS2. To model authentic infection, physiologically relevant cellular systems are essential. We therefore employed polarized HepG2 hepatocytes and highly transfectable 293T cells, as conventional A549 alveolar cells lack significant ACE2 expression [6]. To evaluate our compound in a living system, we utilized a murine model. While wild-type mice are not fully permissive to SARS-CoV-2 due to species-specific differences in ACE2, this model allowed us to directly assess the compound’s ability to modulate the expression of the target host proteins in vivo. Our results demonstrate that oral administration of EMC significantly downregulated ACE2 and TMPRSS2 expression in hepatic and renal tissues, as confirmed by immunohistochemical quantification. Importantly, this effect was achieved without inducing weight loss or histopathological damage, indicating an excellent safety profile [10]. These findings collectively position EMC as a safe, nutritionally derived modulator of viral entry machinery with prophylactic or adjunctive therapeutic potential.

Natural products serve as a key resource for safe, effective antivirals. Plant compounds offer clinical benefits in COVID-19 by restricting viral entry/replication and reducing inflammation/oxidative stress, which drive severe progression [11]. MC, used as a vegetable and medicine in the tropics, has historical applications for diabetes, infections, skin disorders, gout, and parasites [12]. Recent studies confirm antidiabetic, lipid-lowering, antibacterial, and antiviral (anti-HIV) effects via bioactive constituents. Chronic inflammation and oxidative stress link metabolic syndrome and severe viral disease, making MC’s antioxidant/immunomodulatory properties ideal for host-directed SARS-CoV-2 therapy. The 80% ethanolic MC extract showed the strongest antioxidant activity, mainly from ascorbic acid, quercetin glycosides, and cucurbitane triterpenoids. SARS-CoV-2 disrupts redox homeostasis, causing inflammation and RAAS imbalance. Severe cases feature elevated cytokines (TNF-α, IL-1β, IL-6, IL-8) and oxidative markers, with reduced antioxidants [17]. MC extracts suppress NF-κB and cytokine production. In LPS-stimulated RAW264.7 cells, they reduced TNF-α release, downregulated IL-1α/β/TNF-α expression, and inhibited MAPK [36,37]. Critical patients exhibit surges in IL-7, IL-10, MCP-1, G-CSF, MIP-1α, indicating cytokine storm leading to ARDS, multi-organ failure, and death [38]. Findings suggest MC as a safe plant agent for restoring redox balance and attenuating hyperinflammation in COVID-19.

The EMC is enriched in polyphenolics—notably *p*-coumaric acid, rutin, and quercetin—that underpin its multifaceted bioactivity. *p*-coumaric acid exhibits high-affinity binding to the SARS-CoV-2 main protease (M^pro^) catalytic dyad, while also engaging the spike RBD and host ACE2 interface, suggesting dual interference with viral entry and proteolytic maturation [39,40]. Concurrently, *p*-coumaric acid potently suppresses canonical inflammatory cascades (NF-κB, MAPK) and abrogates the secretion of key cytokines (TNF-α, IL-6, IL-1β) that orchestrate the COVID-19 cytokine storm [38,41]. Its robust radical-scavenging and Nrf2-activating capacity further counteracts SARS-CoV-2-induced oxidative burst, thereby preserving endothelial integrity and mitigating redox-driven tissue injury [37]. Collectively, these orthogonal antiviral, anti-inflammatory, and antioxidant mechanisms position *p*-coumaric acid—and by extension EMC—as a promising multifunctional nutraceutical scaffold for host-directed therapy against SARS-CoV-2 and related inflammatory viral syndromes.

Rutin is not just another flavonoid—it is rapidly emerging as one of the most versatile natural compounds against COVID-19. Computer models have consistently demonstrated that they are capable of locking tightly onto the virus’s main protease and can also jam the spike protein or block ACE2, potentially stopping the virus before it even enters cells [41,42]. Once infection is underway, rutin slams the brakes and shuts down two major inflammatory pathways (NF-κB and NLRP3), dramatically cutting the number of the exact cytokines—IL-6, IL-1β, TNF-α—that drive deadly cytokine storms and organ damage. At the same time, its powerful antioxidant ability allows it to mop up the oxidative destruction caused by the virus while protecting blood vessels, preventing dangerous clots, and stabilizing mast cells [43]. In short, it is a single, safe, inexpensive, widely available plant compound that mitigates nearly every major problem in severe COVID-19—viral growth, inflammation, oxidation, clotting, and endothelial injury—making it an ideal candidate for real-world prevention and early treatment [44].

Quercetin, a ubiquitously distributed flavonol, exhibits pleiotropic pharmacology, encompassing antiviral, anti-inflammatory, antioxidant, immunomodulatory, and vasculoprotective effects, with established safety levels at oral doses of ≤1 g/day in humans [45]. Its broad-spectrum antiviral activity spans hepatitis C virus (HCV), hepatitis B virus (HBV), avian influenza virus (IAV), dengue virus (DENV), human immunodeficiency virus (HIV), and Ebola virus. Mechanistically, quercetin directly scavenges reactive oxygen species (ROS), activates Nrf2-antioxidant response element (ARE) signaling, and suppresses NF-κB-driven transcription of proinflammatory cytokines while attenuating Th2 skewing and innate immune hyperactivation. In SARS-CoV-2 models, quercetin inhibits viral entry into Vero E6 cells and displays exceptional in silico binding to 3-chymotrypsin-like protease (3CL^pro^) and RNA-dependent RNA polymerase (RdRp), hindering polyprotein processing and genome replication [43]. By integrating direct antiviral interference with potent suppression of NF-κB-mediated cytokine release and oxidative stress mitigation, quercetin emerges as a multifunctional, orally bioavailable nutraceutical uniquely poised to simultaneously restrain viral propagation and ameliorate the hyperinflammatory and redox-driven pathology characteristic of severe COVID-19.

Taken together, the bioactive trio of *p*-coumaric acid, rutin, and quercetin endows MC with exceptional antioxidant and anti-inflammatory potency directly relevant to COVID-19 pathophysiology. These compounds collectively scavenge reactive oxygen species, suppress NF-κB- and NLRP3-driven cytokine storms, and interfere with SARS-CoV-2 protease and entry machinery. Given its centuries-long record of safe human consumption, MC represents a promising, accessible complementary intervention capable of reducing the viral burden, moderating hyperinflammation, and accelerating recovery in COVID-19 patients.

## 4. Materials and Methods

### 4.1. Materials

*M. charantia* L. specimens were collected from Taichung, Taiwan, in 2024, and taxonomically authenticated by Professor Guan-Jhong Huang at China Medical University. A voucher specimen was deposited in the Department of Chinese Pharmaceutical Sciences and Chinese Medicine Resources at China Medical University (Accession No. CMU-202401). Bitter melon fruits (1 kg) were air-dried in the shade, ground into a fine powder, and subjected to extraction using 95% ethanol at a 1:4 (*w*/*v*) ratio. The extraction process was carried out at 65 °C for 2 h. The resulting extract was filtered twice and concentrated under reduced pressure to obtain a viscous residue, which was subsequently freeze-dried for 3 days to remove residual moisture and stored at −20 °C until use.

### 4.2. Determination of p-Coumaric Acid, Rutin, and Quercetin by HPLC

The chemical composition of EMC was characterized by high-performance liquid chromatography (HPLC) using a Hitachi Ltd. system (Tokyo, Japan) equipped with a TSKgel ODS-80Tm reverse-phase column (250 × 4.6 mm i.d., 5 µm particle size; Tosoh, Yamaguchi, Japan). Eluted fractions were identified based on their retention times relative to authenticated reference standards, with component identities further validated by comparing UV absorption spectra at 254 nm using a photodiode array (PDA) detector. Chromatographic separation was achieved with a mobile phase comprising 0.4% acetic acid in water (solvent A) and methanol (solvent B), employing the following linear gradient: 0 min, 100% A; 0–2 min, 99% A/1% B; 2–4 min, 97% A/3% B; 4–8 min, 95% A/5% B; 8–10 min, 90% A/10% B; 10–20 min, 75% A/25% B; 20–30 min, 65% A/35% B; 30–40 min, 50% A/50% B; 40–50 min, 30% A/70% B; 50–60 min, 100% A. The flow rate was maintained at 2.0 mL/min, with an injection volume of 20 µL. All analyses were performed at ambient temperature to ensure reproducibility and precision. High-purity standards (≥98%) of *p*-coumaric acid (C9008), rutin (R5143), and quercetin (Q4951) were supplied by Sigma-Aldrich (St. Louis, MO, USA) and used as reference compounds in this study. These phenolic compounds were confirmed by comparing their retention times and UV spectra with those of authentic standards. Calibration curves were established from the relationship between peak-area ratio (y) and concentration (x, μg/mL), yielding the following regression equations and correlation coefficients (*r*^2^): *p*-coumaric acid, y = 227403x − 212876 (*r*^2^ = 0.993); rutin, y = 44895x − 30495 (*r*^2^ = 0.993); quercetin, y = 19397x − 16036 (*r*^2^ = 0.997).

### 4.3. Cell Culture and Treatment

HepG2 and 293T cell lines, pivotal models for studying liver and kidney cellular responses, were procured from the Bioresource Collection and Research Center in Hsinchu, Taiwan. These cells were meticulously cultured in Dulbecco’s Modified Eagle Medium (DMEM) supplemented with 10% fetal bovine serum (FBS), maintained precisely at 37 °C under a 5% CO_2_ atmosphere to mimic physiological conditions. For experimental assays, 2.5 × 10^4^ cells were carefully seeded into six-well plates, allowed to adhere and proliferate for 24 h to establish a stable monolayer, and subsequently subjected to treatments as dictated by the experimental protocol, ensuring consistent and reproducible conditions for downstream analyses.

### 4.4. Cell Viability

HepG2 and 293T cells were meticulously seeded into 96-well plates at a density of 2.5 × 10^4^ cells per well, ensuring optimal conditions for proliferation. Following a 24 h exposure to serial concentrations of EMC (50, 100, 250, and 500 μg/mL), *p*-coumaric acid (6.25, 12.5, 25, and 50 μM), rutin (6.25, 12.5, 25, and 50 μM), and quercetin (6.25, 12.5, 25, and 50 μM), cells were subsequently incubated with the MTT reagent (3-(4,5-dimethylthiazol-2-yl)-2,5-diphenyltetrazolium bromide, HY-15924; (Med-ChemExpress, Monmouth Junction, NJ, USA)), a standard colorimetric marker of cellular metabolic activity. After a 3 h incubation, during which viable cells reduced the yellow MTT to purple formazan crystals, the optical density was quantified at 570 nm using a high-precision ELISA microplate reader (Molecular Devices, San Jose, CA, USA). Measurement of formazan generation provided a quantitative index of cell viability and proliferative capacity across treatment groups, offering an essential metric for determining the cytotoxic or protective potential of EMC, *p*-coumaric acid, rutin, and quercetin. The experiments were repeated at least three times, and each concentration was tested in triplicate in each experiment (*n* = 3). As a control, cells were treated with an equal volume of PBS as a vehicle.

### 4.5. Western Blot Analysis

Protein extraction from cells or tissue samples was performed using RIPA buffer (GENESTAR, Kaohsiung, Taiwan), followed by centrifugation at 10,000× *g* for 10 min at 4 °C to isolate soluble proteins in the supernatant. Total protein content was quantified using the Bio-Rad protein assay kit (Bio-Rad, Hercules, CA, USA) and the Bradford method for precise colorimetric measurement. Equivalent protein quantities were separated via SDS-PAGE (100 V, 90 min) and transferred onto PVDF membranes at 200 mA for 2 h. Following blocking with 5% bovine serum albumin (BSA) in TBST, membranes were incubated overnight at 4 °C with primary antibodies against ACE2 (GTX101395, 1:500) and TMPRSS2 (GTX100743, 1:500) (GeneTex, San Antonio, TX, USA). Membranes were then incubated with HRP-conjugated goat anti-rabbit IgG (ARG65351, 1:5000; Arigo, Hsinchu, Taiwan) for 1 h. Protein bands were detected using ECL substrate (Merck, Branchburg, NJ, USA) and quantified using Kodak Molecular Imaging Software 5.0 (Kodak, New York, NY, USA) for densitometric analysis of ACE2 and TMPRSS2 expression.

### 4.6. Mouse Model

Female C57BL/6 mice, aged 6–8 weeks and with body weights ranging from 18 to 20 g, were obtained from BioLASCO Taiwan Co. (Taipei, Taiwan) and acclimatized in a controlled environment (22 ± 2 °C, 55 ± 10% humidity, 12 h light/dark cycle) with free access to standard chow and water. Mice were randomly assigned to two experimental groups (*n* = 5 per group): Group I, the normal control, received distilled water by oral gavage; Group II was treated with EMC proportional to 0.1 g/kg body weight, administered daily via oral gavage for 10 days to evaluate its physiological effects. Body weights were measured on days 0, 3, 5, and 10 using a calibrated digital scale to assess potential treatment-related changes. On day 14, mice were humanely euthanized, and the liver, kidney, and lung tissues were harvested for further analysis. All study protocols were conducted in accordance with the Institutional Guidelines of the China Medical University for the Care and Use of Experimental Animals (IGCMU-CUEA) and were approved by the Institutional Animal Care and Use Committee of the China Medical University (IACUC-CMU; Taichung, Taiwan; Protocol no. CMUH-IACUC-SN2025-209). All procedures were performed in accordance with the institutional guidelines for the care and use of laboratory animals, which are established based on and fully consistent with internationally accepted principles, including the ‘3Rs’ (Replacement, Reduction, and Refinement).

### 4.7. Histopathological Analysis

Liver, kidney, and lung tissue specimens, fixed in 10% neutral-buffered formalin, were embedded in paraffin and sectioned at a consistent thickness of 4–5 µm using a rotary microtome. The sections were stained with hematoxylin and eosin (H&E) to assess histopathological alterations, following standard protocols. Stained slides were visualized under a Nikon ECLIPSE TS100 light microscope (Nikon Corporation, Tokyo, Japan) equipped with appropriate objectives, and representative images were captured using an integrated digital imaging system to document the morphological changes in detail.

### 4.8. Immunohistochemistry (IHC)

Liver, kidney, and lung tissue specimens were fixed in 10% neutral-buffered formalin, embedded in paraffin, and sectioned at 4–5 µm thickness using a rotary microtome. Immunohistochemical staining was performed by incubating the sections with primary antibodies targeting angiotensin-converting enzyme 2 (ACE2; bs-1004R, Bioss Inc., Woburn, MA, USA; 1:50 dilution) and transmembrane serine protease 2 (TMPRSS2; ab214462, Abcam, Cambridge, UK; 1:200 dilution) overnight at 4 °C, following antigen retrieval and blocking of non-specific binding sites. Visualization and image acquisition were conducted using a Nikon ECLIPSE TS100 light microscope (Nikon Corporation, Tokyo, Japan) equipped with a digital imaging system. Staining intensity was quantitatively analyzed using ImageJ 1.54 software (National Institutes of Health, Bethesda, MD, USA) to assess protein expression levels.

### 4.9. Statistical Analyses

All data are presented as the mean ± standard deviation (SD) to summarize central tendency and variability. Statistical analyses were performed using SPSS software (version 21.0; SPSS Inc., Chicago, IL, USA). For comparisons between two independent groups, an unpaired two-tailed Student’s t-test was employed to assess differences in means. For comparisons involving three or more groups, one-way analysis of variance (ANOVA) was conducted, followed by Scheffé’s post hoc test to identify specific group differences while controlling for Type I errors. Statistical significance was defined at a *p*-value threshold of <0.05, indicating a less than 5% probability of observing the results by chance.

## 5. Conclusions

The present study comprehensively demonstrates that EMC, in conjunction with its bioactive components—*p*-coumaric acid, rutin, and quercetin—exerts a pronounced inhibitory effect on the expression of ACE2 and TMPRSS2 across cellular and tissue models. By downregulating these key host proteins, which facilitate SARS-CoV-2 entry through spike protein binding to ACE2 and TMPRSS2-mediated priming of the viral spike protein for membrane fusion, these compounds likely impede viral attachment and internalization. This suppression of ACE2 and TMPRSS2 expression suggests a potential mechanism by which EMC and its constituents may attenuate SARS-CoV-2 replication and infectivity, offering a promising avenue for therapeutic intervention against COVID-19.

## Data Availability

The original contributions presented in this study are included in the article. Further inquiries can be directed to the corresponding author.
